# Does experienced seclusion or restraint affect psychiatric patients’ subjective quality of life at discharge?

**DOI:** 10.1186/1752-4458-7-28

**Published:** 2013-12-05

**Authors:** Päivi Soininen, Hanna Putkonen, Grigori Joffe, Jyrki Korkeila, Pauli Puukka, Anneli Pitkänen, Maritta Välimäki

**Affiliations:** 1 Hospital District of Helsinki and Uusimaa, Hyvinkää Hospital Area, Tuusula, Finland; 2 Faculty of Medicine, Department of Nursing Science, University of Turku, Turku, Finland; 3 Faculty of Medicine, Psychiatry, University of Turku, Turku, Finland; 4 Hospital District of Southwest Finland, Turku, Finland; 5 National Institute for Health and Welfare, Turku, Finland; 6 Pirkanmaa Hospital District, Tampere, Finland; 7 Department of Psychiatry, Helsinki University Central Hospital, Helsinki, Finland; 8 Vanha Vaasa Hospital, Vaasa, Finland

**Keywords:** Quality of life, Seclusion, Restraint, Schizophrenia, Mood disorders

## Abstract

**Background:**

In Finland major effort has been invested in reducing the use of coercion in psychiatric treatment, and the goal is to diminish the use of coercion by 40% by 2015. Improving patients’ quality of life (QoL) has gained prominence in psychiatric treatment during the past decade. Numerous studies have shown that most secluded or restrained patients (S/R patients) would prefer not to have had this experience. Experience of S/R could affect negatively patients’ QoL, but empirical data on this issue are lacking.

**Aim:**

The study aimed to explore the effect of experienced S/R on the subjective QoL of psychiatric in-patients.

**Method:**

This study explored subjective QoL of the S/R patients. At discharge, S/R patients completed the Short Form of the Quality of Life Enjoyment and Satisfaction Questionnaire (Q-LES-Q-SF).

**Results:**

We found that S/R patients’ (n = 36) subjective QoL was significantly better than that of non-S/R patients’ (n = 228). Most non-S/R patients were diagnosed with mood disorders (mostly depression). Most of S/R patients were diagnosed with schizophrenia, schizotypal and delusional disorders. The mean duration of S/R was 2.3 days, median was one day and mean length of the hospitalization after S/R episode was 2.5 months.

**Conclusion:**

Our cross-sectional findings suggest that S/R does not considerably influence patients’ QoL or that the influence is short-lived. Because baseline QoL was not measured this remains uncertain. There are also many other factors, such as negative mood, which decrease the patients’ QoL ratings. These factors may either mask the influence of S/R on QoL or modify the experience of QoL to such an extent that no independent association can be found at the time of discharge.

## Introduction

Coercion in the Finnish legislation is defined as involuntary admission to observation and treatment in psychiatric hospitals, treatment against a person’s own will, and special limitations, for example, as regards forcible holding, isolation or seclusion, restraint or tying down, and limitations of contacts [1]. In Finland major effort has been invested in reducing the use of coercion in psychiatric treatment, and the goal is to diminish the use of coercion by 40% by 2015 [2,3]. This is in line with international ethical guidelines [4-7].

Quality of life (QoL) has been recognized as an important outcome of psychiatric treatment [8,9]. QoL includes a philosophical dimension and refers to “good life” [8]. This covers physical, emotional, mental, social and behavioural components of well-being [10]. Although QoL stems primarily from subjective experience, it also has an objective aspect, such as social functioning, living conditions, education, employment, finance, housing and leisure activities [11,12]. Although there is no strict and universally accepted definition for QoL, many researchers agree that patients’ statements on satisfaction together with daily functioning are relevant indicators of perceived QoL [12,13]. The WHO QoL group states that the QoL is an individual’s perception of his or her position in life in the context of the culture and value system in which he or she lives, in relation to his or her goals, expectations, standards and concerns [14].

Murphy & Murphy [11] compared the QoL of mentally ill people with that of individuals without mental illness, exploring the role of self-esteem, self-efficacy and social functioning and found that individuals with mental illnesses had poorer ratings on all aspects of QoL than the healthy subjects. Similar results have been reported by Tompenaars et al. [15], who explored relationships between social functioning and QoL. Saarni et al. [8] found that individuals with schizoaffective disorders had the lowest well-being scores on all QoL measures used. In the same study schizophrenia and bipolar disorder patients’ subjective QoL was higher than the estimation of patients’ relatives and stakeholders. Further, current depressive symptoms explained most of the loss of QoL [8,16]. Goppoldova et al. [10] assessed and compared subjective QoL among three major psychiatric diagnostic categories (psychosis, mood, anxiety disorders) on admission and discharges from hospitalization and found that on admission psychotic patients’ subjective QoL was highest and showed the least improvement during hospitalization. The greatest improvement during hospitalization in subjective QoL was seen in patients with depression and the greatest deterioration of QoL during hospitalization in patients with anxiety problems [10].

Coerced patients’ experiences of their treatment and perceived quality of life [17] are a central issue when promoting and providing comprehensive and effective mental health services and interventions [18]. In many countries seclusion and restraint (later S/R) are used to manage disruptive and violent behaviour of psychiatric inpatients as a last resort to ensure their own or others safety [19,20]. These measures have remained controversial since they are likely to cause distress and emotional trauma to both patients and staff [21,22]. Despite the fact that patients most often consider S/R unnecessary and punitive, the use of S/R is sometimes felt to offer some benefit [23,24]. Although S/R is suggested to prevent injury and reduce agitation, documented efficacy, effectiveness and safety of these interventions are still lacking [25,26]. Hence the use of these measures is often in doubt [27,28].

Given patients negative perceptions of S/R, one could argue that S/R may impair patients’ QoL, although empirical data on this issue are lacking. The authors were able to locate a number of studies on the QoL of patients treated in restricted treatment environments in psychiatric hospitals [12,29-32] but not a single study focused on the QoL of S/R patients.

The study aimed to explore the effect of experienced S/R on the subjective QoL of psychiatric in-patients by comparing during hospitalization S/R patients’ subjective QoL with non-S/R patients’ QoL. Second the study aimed to assess possible associations of demographic and clinical variables with QoL. The third aim was to assess a possible association of length of stay following S/R and QoL, and the association of S/R duration and QoL. In the light of earlier studies [21,24,33,34] we assumed that S/R patients’ QoL was poorer than that of patients without S/R experience.

## Method

### Ethics

The study was evaluated by the Ethics Committee of the Hospital District of Helsinki and Uusimaa and approved by appropriate institutional authorities. After a complete description of the study, participants gave written informed consent. Participation was voluntary and data were treated in confidence [35]. It was emphasized that participation or refusal would not affect treatment.

### Setting

Data were collected over one year 2009–2010 on five acute psychiatric wards of the Kellokoski Hospital. The hospital serves as a local psychiatric facility for an area of 180 000 inhabitants and in addition, as a specialized institution for forensic and difficult-to-manage patients for a district of 1,500 000 inhabitants. The acute wards treated adult patients and had a seclusion room. The first of the acute closed wards was an admission ward and had a mean length of stay of two weeks. This ward had the highest number of seclusion and restraint events among the acute wards per year (in 2008, 99 seclusion and 15 restraint events). The second ward treated patients with mood disorders and rarely used seclusion or restraint and was chosen as a study ward for this reason (to enable comparison with non-S/R patients’ QoL and S/R patients’ QoL). The third ward treated acute patients diagnosed with schizophrenia, bipolar disorders etc. (in 2008, 28 seclusion and two restraint events). The fourth ward treated mostly first-episode psychotic (FEP) patients (in 2008, 14 seclusion and four restraint events) and the last one rehabilitated patients with schizophrenia (in 2008, 18 seclusion and two restraint events). All study wards had 12–20 beds and were operational 24 hours seven days a week.

### Patients

Patients were included if they were: aged 18–65, discharged to home (i.e. not transferred to other wards or hospitals) and fluent in Finnish. Respondents’ characteristics (age, gender, diagnosis, marital status, socioeconomic situation, living arrangements, number of hospitalizations) and (for S/R patients) S/R duration and time span from S/R episode to discharge were collected from the patients’ hospital records by the researcher (PS).

Before data collection, a researcher gave written and oral information to the staff of each ward. Every ward manager delegated nurses to be responsible for data collection on the ward. When the decision on a patient’s discharge was made, each patient received complete information on the study in written and oral form and gave written informed consent. Patients were informed that their participation was voluntary and that refusal would not affect their treatment. Those who gave written consent independently completed the Q-LES-Q SF questionnaire using paper and pencil.

### Instrument

The instrument used in the present study was a structured self-report questionnaire, the Quality of Life and Satisfaction Questionnaire [36]. In this study the 16-item short version (Q-LES-Q- SF) was used [37]. This scale has previously been used among patients with severe mental health problems, also measuring inpatients QoL [38,39]. Out of the 16 items, 14 items measure patients’ general activities, such as social relationships, emotions, physical health, living conditions and housing situations (patients’ subjective QoL). The remaining two items cover overall satisfaction and medication during the past seven days. Each question is scored on a 5-point Likert scale (1 = very poor, 5 = very good degree of enjoyment or satisfaction). The global QoL index is summed from the first 14 items. Q-LES-Q has shown acceptable psychometric properties when measuring QoL in patients with schizophrenia [38,39]. The patients completed Q-LES-Q-SF after the decision but prior to discharge.

### Sample

**Figure 1:**
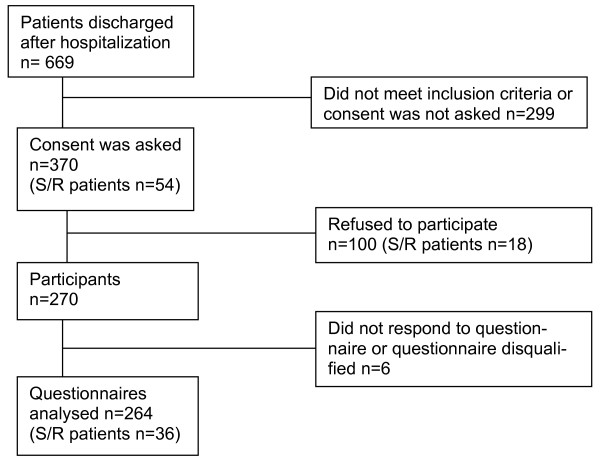
Flow chart of recruitment to the study of S/R and non-S/R patients’ QoL.

During the data collection period, a total of 669 patients were discharged from the study wards according to the hospital statistics (no information on S/R patients). Out of 669 patients 370 were asked to participate in the study (54 of these were S/R patients). One hundred refused (18 of these were S/R patients) mainly on the acute admission ward. The remaining 270 patients (73%) agreed to participate. Six patients did not return the questionnaire or the response was disqualified due to inadequate responses. Finally 264 questionnaires were analysed. The remaining 299 patients (44.7%) did not fulfil the inclusion criteria or were not invited to participate due to quick discharge, work overloading of the staff, insufficient information on the deputy staff or other reasons (Figure 1).

The respondents’ ages ranged from 18 to 65 (mean = 38, SD = 12.2) and 57% (n = 152) were women. Most of the respondents (n = 112, 46.5%) were diagnosed (International Classification of Disease, 10^th^ Revision, WHO) with mood disorders (F30-39) followed by the schizophrenia group (F20-29) (n = 87, 36.1%). Of all eligible respondents (n = 264), 36 patients (13.6%) had been secluded/restrained during their hospital stay. For respondents’ characteristics (see Table 1).

**Table 1:** Characteristics of the Non-SR and SR patients

	**Non-S/R**		**SR**				
	**(n = 228)**		**(n = 35)**				
	**n**	**%**	**n**	**%**	**χ**^**2**^	**df**	**p**
Age					0.21	1	0.648
– 38 years	111	49	19	53			
39 - years	117	51	17	47			
Gender					1.83	1	0.176
Male	93	41	19	53			
Female	135	59	17	47			
Diagnosis^1^							
F20-29	68	33	19	54	5.87	1	0.015^2^
F30-39	101	49	11	31	3.73	1	0.054^2^
Other	37	18	5	14	0.29	1	0.596^2^
Hospitalization^1^					7.85	2	0.020
Once (first time)	77	38	6	17			
2-5 times	66	32	11	31			
More than 5 times	61	30	18	51			
Marital status^1^					6.36	1	0.012
Marriage/common-law	74	36	5	14			
Single or divorced	132	64	30	86			
Socioeconomic status^1^					0.56	1	0.455
Working/studying	66	32	9	26			
Other	140	68	26	74			
Living^1^					3.81	1	0.051
Family or partner	100	49	11	31			
Other	103	51	24	69			

^1^Non-SR patients, n = 206; SR patients, n = 35.

^2^Diagnosis vs. all others.

The duration of an S/R episode was from two hours to 336 hours (mean = 57.22, MD = 25.50, SD = 82.06) and time span from S/R episode to discharge ranged from one day to 285 days (mean = 81.85, MD = 57.70, SD = 74.84). The S/R episodes occurred most often at the beginning of the hospital stay. Reasons for S/R were as follows: patient’s suicidal (n = 3), aggressive, threatening, or psychotic behaviour (n = 27), or noisy or threatening behaviour that disrupted other patients’ treatment (n = 5).

### Data analysis

Descriptive analyses (frequency, percentage, mean, standard deviation, median) were performed for individual items of the Q-LES-Q SF. Then the subscale item-scores 1–14 were summed according to the instructions of the developer of the instrument [37]. Shapiro-Wilk test was used to test the normality of the distribution of variables. Cross-tabulations with Chi Square tests were used to analyse possible differences between S/R patients and non-S/R patients regarding their background information. Independent samples t-test was used to test the statistical significance of differences between S/R patients and non-S/R patients in their evaluations of subjective QoL regarding the total subscale and individual items of the instrument. Interaction between coercion (S/R, non-S/R) and background variables was evaluated by two-way analysis of variance. Due to some skewed distributions the results of the statistical tests were checked by nonparametric methods when appropriate. P-values of 0.05 or less were regarded as statistically significant. To control for the imbalanced background factor distribution, a non-S/R patients’ sample was selected for the S/R patients matched for age, gender, ward and diagnosis.To replicate the findings in comparable samples a matched non- S/R patients sample was chosen for the S/R patients. The matching criteria were age, gender, ward and diagnosis. In case of several matching patients random selection was applied.

## Results

### S/R and non-S/R patients’ QoL

Of all responses analysed (n = 264), 228 (82.4%) responses came from non-S/R patients and 36 (13.6%) responses came from S/R patients. S/R patients’ QoL (mean = 70.62, SD = 12.64, range 41–100) was significantly higher than that of non-S/R patients (mean = 55.29, SD = 19.22, range 5–100) on the subscale (t = 6.2, DF = 61.8, p < 0.001). When the items were analysed separately, the ratings in all items except living arrangements, vision and medication were higher among S/R patients than non-S/R patients. Item 11 concerning living arrangements had the largest number of missing responses (see Table 2).

**Table 2:** Inpatients Qol (Q-LES-Q-SF) after discharge decision, differences of mean ratings between S/R and non-S/R-patients

	**S/R**	**Mean**	**Non-S/R**			
	**N**	**(SD)**	**N**	**Mean (SD)**	**t**	p-value*
Physical health	36	3,97 (0,84)	227	3,33 (1,07)	4.09	<0.001
Feelings	36	3,86 (0,83)	227	3,19 (1,19)	4.18	<0.001
Work	27	3,52 (1,08)	206	2,68 (1,17)	3.50	0.001
Household duties	34	3,71 (0,83)	219	2,93 (1,15)	4.75	<0.001
Social relationship	36	3,97 (0,73)	225	3,29 (1,18)	4.76	<0.001
Family relationship	35	4,11 (0,79)	221	3,62 (1,19)	3.13	0.003
Leisure activities	35	3,71 (0,96)	223	2,96 (1,13)	3.75	<0.001
Daily functioning	36	3,94 (0,86)	227	3,10 (1,12)	5.22	<0.001
Sexual drive/performance	35	3,66 (0,96)	220	2,87 (1,21)	3.36	<0.001
Economic status	36	3,14 (1,09)	226	2,59 (1,17)	2.62	0.009
Living/arrangements	29	3,72 (1,03)	167	3,44 (1,14)	1.24	0.217
Mobility	36	4,36 (0,76)	224	3,99 (0,99)	2.17	0.031
Vision	36	4,17 (0,84)	223	3,86 (1,04)	1.69	0.092
Overall wellbeing	36	3,81 (0,85)	224	3,10 (1,14)	4.37	<0.001
Medication	34	3,82 (0,93)	219	3,55 (0,98)	1.53	0.127
Overall satisfaction	36	4,00 (0,86)	225	3,20 (1,08)	4.25	<0.001

*Independent sample t-test.

Out of all 264 participants 52% of non-S/R patients were treated on the admission ward and 80% of S/R patients were treated on other acute wards. Most of the S/R patients who refused to participate in the study were treated on the admission ward. The differences in the Q-Les-Q scores remained after comparison of samples matched for age, gender, ward and diagnosis. S/R patients’ (n = 30) QoL (mean = 71.05, SD = 12.86, range 41.07-100) was significantly (p = 0.019) higher than non-S/R patients’ (n = 30) QoL (mean = 59.80, SD = 22.01, range 5–92.85). When items were analysed separately, the ratings in feelings (p = 0.019), work (p = 0.025), daily functioning (p = 0.016), sexual drive (p = 0.004) and vision (p = 0.03) were significantly higher in the S/R group than in the non-S/R group.

### S/R and non-S/R patients’ background factors and association with QoL

We compared associations between background factors (age, gender, diagnoses, month of hospitalization, marital, socioeconomic status and living arrangements) and QoL ratings in S/R and non-S/R patients and found that S/R patients’ ratings were significantly better than those of non-S/R patients in almost all subgroups. Among first episode psychoses (FEP) who belonged to the “other diagnoses” group, there were no statistically significant differences in QoL ratings between S/R and non-S/R cases nor did the respective QoL ratings differ with respect to marital status. Among patients hospitalized for the first time and those living in a relationship and marital status single, no significant differences were found between the QoL ratings of S/R and non-S/R patients. There was no significant interaction between background factors and coercion (S/R, non-S/R), see Table 3.

**Table 3:** Association between background factors and QoL ratings (Percent Max Score) among Non-SR and SR patients

	**Non-SR patients**			**SR patients**				**Total**		
**Age**	**n**	**Mean**	**Std Dev**	**n**	**Mean**	**Std Dev**	**p (non-SR vs. SR)**	**n**	**Mean**	**Std Dev**
**– 38**	111	53,56	17,22	19	67,72	10,10	<0.0001	130	55,63	17,10
**39 -**	113	57,00	20,95	16	74,08	14,72	0.0021	129	59,12	21,01
p (age)		0.18			0.16				0.096	
**Gender**	**n**	**Mean**	**Std Dev**	**n**	**Mean**	**Std Dev**		**n**	**Mean**	**Std Dev**
**female**	132	55,94	20,14	17	72,13	12,35	<0.0001	149	57,79	20,05
**male**	92	54,37	17,91	18	69,21	13,12	0.0011	110	56,80	18,03
p (gender)		0.55			0.50				0.45	
**Hospitalizations**	**n**	**Mean**	**Std Dev**	**n**	**Mean**	**Std Dev**		**n**	**Mean**	**Std Dev**
**1 - 3**	140	54,53	18,52	16	69,15	10,38	<0.0001	156	56,02	18,38
**4 - 5**	60	56,93	20,24	18	73,09	13,83	0.0022	78	60,66	20,08
p (hosp)		0.41			0.36				0.30	
**Hospitalizations**	**n**	**Mean**	**Std Dev**	**n**	**Mean**	**Std Dev**		**n**	**Mean**	**Std Dev**
**1**	75	56,34	17,75	6	67,67	15,35	0.13	81	57,18	17,75
**other**	125	54,59	19,80	28	72,00	11,75	<0.0001	153	57,78	19,75
p (hosp)		0.63			0.44				0.64	
**Marital**	**n**	**Mean**	**Std Dev**	**n**	**Mean**	**Std Dev**		**n**	**Mean**	**Std Dev**
**1 + 3⌐**	128	54,11	19,14	29	71,45	12,07	<0.0001	157	57,31	19,24
**2˜**	74	57,83	18,84	5	70,00	15,12	0.16	79	58,60	18,78
p (marital)		0.18			0.97				0.20	
**Socioeconomic**	**n**	**Mean**	**Std Dev**	**n**	**Mean**	**Std Dev**		**n**	**Mean**	**Std Dev**
**1**^**n**^	63	55,93	16,63	9	73,26	11,11	0.0035	72	58,09	16,99
**2º**	139	55,26	20,13	25	70,51	12,85	<0.0001	164	57,58	19,94
p (socio)		0.82			0.57				0.72	
**Arrangement**	**n**	**Mean**	**Std Dev**	**n**	**Mean**	**Std Dev**		**n**	**Mean**	**Std Dev**
**1‡**	100	53,89	19,38	23	71,17	10,78	<0.0001	123	57,12	19,27
**2†**	99	56,66	18,88	11	71,37	15,63	0.014	110	58,13	19,04
p (arr)		0.31			0.97				0.31	
**Diagnosis₫**	**n**	**Mean**	**Std Dev**	**n**	**Mean**	**Std Dev**		**n**	**Mean**	**Std Dev**
**F20-29**	66	58,13	21,53	18	73,58	12,20	0.0002	84	61,44	20,84
**F30-39**	99	56,00	15,93	11	72,07	10,97	0.0015	110	57,60	16,21
**other**	37	49,30	21,21	5	60,95	12,55	0.24	42	50,69	20,61
p (diagn)		0.07			0.12				0.011	

⌐Marriage or cohabitation.

˜Single.

^n^working.

ºunemployed, pension.

‡with family.

†alone.

**₫** F20-29 Schizophrenia, schizotypal and delusional disorders.

F30-39 Mood (affective) disorders.

### Association of length of stay following S/R and QoL and the association of S/R duration and QoL

There was no statistically significant interaction between time from S/R episode to discharge and also none between S/R duration and QoL variables.

### QoL and ward

The admission ward differed significantly from the other wards (p < 0.001).

## Discussion

Many statements and guidelines [18] recommend taking heed of patients’ opinions and wishes when making decisions on treatments and planning treatment even in situations when restrictions on patients’ care was considered, mainly in situations when patients’ behaviour threatens their own safety of that of others. It is important to consider the effect of the S/R decision on inpatients’ QoL.

### S/R and non- S/R patients’ QoL

Although patients consider S/R unnecessary [24] and more of a punishment than treatment [40], the results of our research indicate that experiencing S/R was associated with improved subjective QoL. The study hypothesis of a long-term negative effect of S/R on QoL was not supported. All respondents were satisfied with their medication, vision and living arrangements at the end of their hospital care. It is noteworthy that mood disorders were associated with poorer QoL ratings, as one could expect. Depressed mood may mask the influence of S/R on QoL or may, as well as background factors, modify the experience of QoL to such an extent that no independent explanation can be found at the time of discharge. The study design did not allow for surfacing of subjective (vs. actual) experience of coercion.

The main diagnostic group among the non-S/R respondents was mood disorders (49%), mostly depression, followed by schizophrenia (33%). Among the S/R patients, the most common diagnosis was schizophrenia (54%) followed by mood disorders (31%), mostly bipolar disorders which concurs with other studies [26,41] and with the national statistics [3]. According to the literature, patients with depression and bipolar disorders have poorer subjective QoL [16] than those with schizophrenia [16]. The higher proportion of mood disorders among the non-S/R patients in this study might, thus, explain their lower QoL as compared to that of the S/R patients. However, the same difference in the QoL in favour of S/R patients remained after comparison of samples matched for background factors including diagnosis. Hospitalization may also improve patients’ subjective QoL in all major diagnosis categories [10] – a phenomenon that could with time attenuate the presumably negative immediate impact of S/R on patient’s QoL.

Adaptation to the illness and treatment system as well as experiences of earlier coercive measures has been shown to influence patients’ perception of coercive measures [42,43]. Possibly patients hospitalized more than once were more familiar with the treatment system, adapted to their symptoms to some extent and lowered their expectations of their living conditions and well-being [44]. In our study most of the S/R patients were hospitalized more than once, only six of them were hospitalized for the first time. Hoekstra et al. [42] concluded that the reasons for earlier S/R patients’ positive experiences of coercion were associated with adaptation; learning to live with the experience rather than assimilation; active coping and controlling. Melle et al. [45] found that FEP QoL improved in two years of follow-up likely due to adaptation [45]. We may assume that the patients experience a genuine advantage of isolation and get a feeling of safety that way. Hopefully the way of treating S/R episodes has been pleasant and respectful towards the patients. However, according to earlier studies this is not always the case [21,24,41].

Patients’ satisfaction with the medication was high in the QoL ratings. The effect of the medication is an important result of the hospital treatment. Unfortunately we did not gather data of medication and this is a limitation of the study. We can assume that quite often patients’ non compliance for the medication was the reason for hospitalization [46].

### Association of QoL and duration of hospital stay

The duration of S/R episodes was quite long mean 2.3 days, yet the median was slightly over one day and care in hospital also lasted quite a long time after S/R episodes (ca. 2.5 months). Patients may have had an opportunity to go over their experiences of S/R episodes and the negative effect of S/R was thus diminished. Mean duration of hospital care in Finland according to official statistics was 34 days [3]. The average time spent in seclusion varies a lot in different study reports; Meehan et al. [40] reported a mean duration in seclusion lasted 2–4 hours [45], Bergk et al. [26] reported mean duration in seclusion from 8.1 hours to 12.3 hours in different groups whereas Stolker et al. [47] reported a median duration of 37 hours in seclusion [47].

### QoL and wards

Other factors during hospitalization may also affected the results – patient’s recovery [48], psycho education received [31,49], therapeutic relationship between nurses and patients [49] or characteristics of the wards. Although all wards were classified in the hospital as acute wards, there were substantial differences in their duty profile. For instance, one of the wards focused in fact on early rehabilitation of subacute patients. Nevertheless, the matched samples comparison did not reveal any substantial effects of wards on the observed improved QoL of the S/R patients. It is noteworthy, however, that some of the patients were secluded/restrained on other wards than those they were discharged from, which made the exploration of the possible ward effect uncertain.

Ristner et al. [12] found that improved QoL among schizophrenic patients was associated with the level of patients’ distress, absence of paranoid symptoms, self-efficacy and self-esteem. To separate these from the direct effect of S/R on the patients’ QoL, the longitudinal study design is needed.

### Strength and limitations

There are a number of strengths and limitations in the study. First, this was the first study to measure subjective QoL among S/R patients. The validity, reliability and feasibility of the Q-LES-Q SF has been assessed in Finland in a study on patients with schizophrenia and related disorders in the acute stage of their illness in hospital care [39]. Nevertheless, we do not know whether this is the most sensitive and specific measure for comparing S/R and non-S/R patients. Furthermore, QoL should be measured in a longitudinal setting at two time-points during the patients’ treatment. – at baseline and endpoint.

Second, the small number of responses among S/R patients may cause bias [50], which may further limit generalization of our results. Third, there emerged a significant disparity in terms of diagnosis and ward allocation between the S/R group and non-S/R group, which could confound the between- group comparison. To eliminate this possible confounding factor, an additional comparison was made for age, gender, diagnosis and ward matched samples. The time from S/R to discharge varied significantly, which may be rather a strength of the study since it makes the results more generalizable.

Fourth, the number of eligible participants who refused to participate was significant (27%). Most of the patients who refised to participate were treated on the acute admission ward, where treatment duration was short and the commitment to the care may have been undermined [51,52]. This is also not surprising since the sample consisted of patients with schizophrenia among whom high refusal and drop out rates are common [53]. At the same time, quite many had no opportunity to participate in the study for various reasons. For example, due to holiday periods the substituting staff may not have been well enough aware of the study project and they did notactively ask the patients’ to participate in the study due to their own limited motivation. However, inlight of our national statistics [3], the study participants represented Finnish inpatients.

Fifth, our aim was to measure subjective QoL of the S/R patients with a structured instrument. Measuring subjective issues is challenging, especially where patients experiences S/R are concerned. We can also ask whether all patients who are being treated behind locked doors experience subjective restrictions during their care. This is an important question and we therefore need to keep in mind that concepts related to patient restrictions always include a variety of debatable connotations.

Sixth, one limitation was that we did not gather the data of the medication.

Future research should focus on 1) separate comparison of QoL within schizophrenia and mood disorder groups; 2) comparison between these two groups, and 3) prospective (i.e., at the beginning and end of hospital treatment) follow-up of the QoL change.

The strength of this study is that despite methodological limitations measuring inpatients’ QoL in the end of hospital care is important and showed that patients with psychotic ideation may benefit more of hospitalization and restrictions than patients with depression. Plans for reducing hospital care and treating patients in outpatient care [2] may have the same or better outcome measured by structured QoL instrument and we can conclude that patients with depression may benefit more from outpatient care more than from hospital care. More studies are needed on how mental health services affect the patients’ QoL.

Due to these methodological limitations this study may be perceived to be more of a pilot study and more studies are needed to make any reliable conclusions about S/R patients’ QoL during their hospital stay. How this should be done in the future should be elicited from those patients themselves using user-driven and patient centred study design.

## Conclusions

According to the findings of this study S/R experience was associated with patients’ better QoL, but this is unlikely to be causal. Whatever the reasons of this finding, the use of S/R as a treatment method cannot be advocated if not absolutely necessary because the vast majority of studies report patients’ negative subjective experiences of S/R. Mentally ill people have poorer QoL than healthy subjects [11,54], and thus improvement in a patient’s QoL should be a goal on psychiatric acute wards. Sometimes isolating a patient from the ward community may enhance that patient’s QoL [31]. Measuring QoL on admission and at discharge would be important so one could estimate the meaning of interventions as S/R measures for QoL.

We can conclude that S/R patients’ long hospitalization and other factors such as rehabilitation, therapeutic relationship, balanced condition, holistic care, adaptation to illness and safe environment may have had an impact on the patients’ QoL. S/R episodes happened mostly just after admission so the change in patients’ condition can be assumed to be remarkable and that leads to better estimations in responses to the QoL instrument.

Patients’ adaptation to the illness and compliance with psychiatric practices raise the question of what kind of treatment should be offered in psychiatric hospitals to improve patients’ QoL where patients’ condition is often psychotic, suicidal or aggressive. In the programmes shortening the hospitalization and increasing outpatient care as well as decreasing the use of coercive measures such as seclusion and restraint are required [2].

### Implications

QoL has been recognized as an important outcome of psychiatric treatment and measuring QoL can improve knowledge of the effectiveness of interventions used in psychiatric treatment. It is difficult to estimate how S/R affects QoL if the time span between intervention and measurement is long. Measuring patients’ QoL with an instrument at the beginning of the hospital care and just after the S/R episode and comparing the change could show what the impact of the intervention was on QoL. Treatment plans should be made together with patients and considering the factors that support QoL in patients’ living (patients’ general activities, social relationships, emotions, physical health, living conditions and housing situations).

More genuine dialog between staff and patients (and relatives) is needed so that seclusion and restraint measures could be prevented and alternatives found. There must be other ways than S/R for patients to get a balance, feel safe and to get staff’s attention and have a say. Patients who perceive S/R positively have admitted the need for these measures. Isolating a patient from the ward milieu some other way; a comfort room, a single room with nurse surveillance, could be examples of alternatives.

## Competing interests

The authors declare that they have no competing interests.

## Authors’ contributions

PS designed the study and involved the data collection, analyses and drafting the manuscript. HP, GJ, JK , and MV were involved in the design of the study, drafting the manuscript and critically reviewed the manuscript. PP was involved in the data analyses and AP was expert in the instrument issue. All authors have read and approved the manuscript.
